# A single visit feeding plate for 3 months old cleft palate infant . A case report

**DOI:** 10.15171/joddd.2017.044

**Published:** 2017-12-13

**Authors:** Amro Mohammed Moness Ali, Abdullah Kamel

**Affiliations:** ^1^Lecturer of Paediatric and Community Dentistry, Paediatric and Community Dentistry Department- Faculty of Dentistry Mania University Egypt; ^2^Assistant Lecturer of Prosthetic Dentistry Prosthetic Dentistry Department Faculty of Dentistry Mania University-Egypt

**Keywords:** Feeding, Cleft palate, Obturators, Infant

## Abstract

Infants with cleft palate (CP) suffer from several difficulties, one of them is feeding, which prevent infant from maintaining adequate nutrition. Usage of feeding plate, special bottles and nipples has been described to overcome this problem. This article describes steps of constructing a single visit feeding plate for a CP infant. The main objective was to provide an infant with a properly functioning feeding prosthesis and to reduce parents’ anxiety originated from multiple dental visits. We can conclude that our feeding plate was successfully achieving the planned objectives.

**Key messages**

Provision of a feeding appliance for a cleft palate patient is challenging, requiring collaborated efforts of a dental team. This work demonstrated the procedural steps for fabrication of a single-visit feeding plate for a cleft palate infant and described the role of both the pedodontist and prosthodontist in such cases.

## Introduction

Cleft lip and palate (CL/P) is one of the most common congenital orofacial anomalies.^1^ Classification, possible etiological factors, pathogenesis and different management protocols of cleft palate have been described in the literature.^2^ Such cases require collaborative efforts by many specialists, among them a pedodontist and a prosthodontist to participate actively and efficiently.^3,4^ Feeding process in a CL/P child is usually difficult and feeding time is very long and both the infant and mother get exhausted.^5^

A feeding plate is not only critical for proper nutrition but also it has a role in craniofacial growth and reduces the incidence of otitis media and nasopharyngeal infections as well.^6^

## Case report

**Figure 1:**
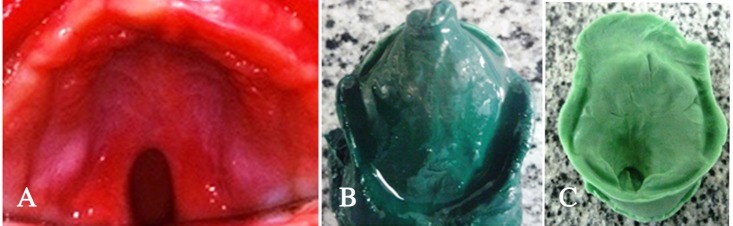
(A) Intraoral view of cleft palate. (B) Primary impression . (C) Primary model

A 3-month-old female infant, with non-contributory medical and family history, was referred to the Paediatric Dentistry Outpatient Clinic, Minia University Dental Hospital, Minia, Egypt, with a chief complaint of difficulty in feeding and nasal discharge. The mother reported that the infant was not able to suckle milk properly even with the use of typical cleft nipples or squeezable bottles and the infant was not gaining proper weight (only 7 pounds). There was no history of previous treatment or surgery for the defect. Intraoral examination revealed a cleft in the uvula, soft palate and secondary hard palate (Veau’s classification: Type II) (Figure 1A).

A detailed examination of the infant, followed by consultation with a prosthodontist, was carried out, and after the parents' approval, the fabrication of a feeding plate was decided on.

## Fabrication of the feeding plate

**Figure 2:**
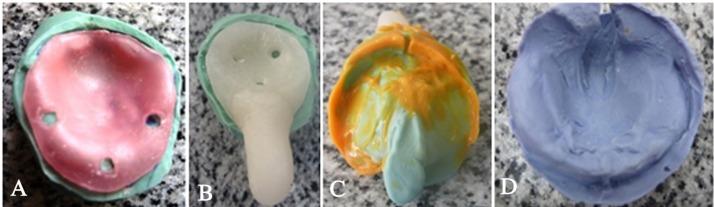
(A) Wax spacer ,(B) Special Tray ,(C) Secondary impression, (D)Master cast

Primary impression was made using low-fusing impression compound (Kerr UK Ltd,Peterborough, UK). First the defect was filled with a piece of Vaseline gauze; then green stick was softened in warm water and kneaded with caution to avoid thermal injury. An alginate spatula was used to carry the impression material into the infant’s mouth and the material was gently pressed against the hard palate and into the buccal and labial vestibules, while the baby was held in prone position in the mother’s lap. During this step the infant was crying. The impression was inspected thoroughly; it had satisfactorily covered all the supporting areas for the feeding plate (Figure 1B). Then a primary model was obtained using heavy putty rubber base (Figure 1C). The primary model was carefully inspected in order to finely determine the borders of the special tray. A 2-mm wax spacer was adapted to the primary model (Figure 2A) and the special tray was constructed with the use of a self-curing fast-setting acrylic resin (Acrostone, WHW, England; Figure 2B). The final impression was made with very high-viscosity condensation silicone rubber base impression material (ZetaplusSpA via bovazecchino, BadiaPolesine [RO], Italy), followed by light-body wash (Figure 2C).

**Figure 3:**
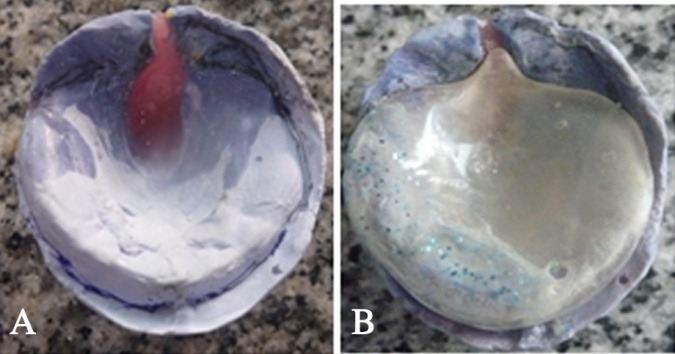
(A) Blocking undercuts , (B) Feeding plate before intraoral checking

The secondary impression was poured with dental stone to obtain a master cast (Figure 2D), blocking out the undercuts with pink wax (Figure 3A); then the plate was fabricated using self-cured acrylic resin (Acrostone, WHW, England). Finally, the edges of this plate were trimmed (Figure 3B).

**Figure 4:**
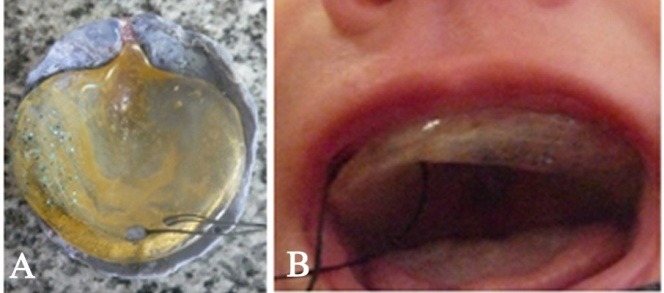
(A)Feeding plate after adjustment and insertion of the silk suture , (B) Intraoral view of the feeding plate

Approximately a 10-inch silk suture (4/0 SINORGMED SILK Shandong ShengmeiMedical supplies Co.,LtD, China) was passed through and tied to the eyelet (made by small acrylic stone) of the feeding plate. The prosthesis was trimmed, finished and polished. Then it was examined in the patient’s mouth; thereafter minor adjustments and final polishing of the feeding plate were carried out (Figure 4A).

The feeding plate was checked in the dental clinic (Figure 4B) and the patient’s mother was asked to feed the baby. Instructions were provided on how to use, clean, function and maintain the feeding appliance. Monthly follow-ups were planned and the mother was informed that the feeding plate could be replaced to accommodate the craniofacial growth before surgical intervention. The infant was gained weight normally during the follow-up period.

The previously described procedure was conducted on the same day the patient was admitted into the hospital.

## Discussion

The prevalence of CL/P in some Egyptian populations ranges from 0.39 to 0.51 per 1000 live births.^7,8^ The main objective during the first months of cleft palate infant’s life is proper weight gain, which results from proper feeding, making the infant ready for future surgical correction.^5,6^ Construction of a feeding appliance not only fills the gap between the nasal and oral cavities, but also it achieves maximum treatment benefits for such patients; at the same time it increases awareness and enhances the skills of diagnosis and management aspects of all the specialists in the interdisciplinary team.^3,4^

Making an impression is the first challenging clinical step in CP infants due to lack of cooperation on behalf of the patient. The oral cavity is too small to be adequate for commercially available impression trays, with a risk of impression material swallowing and aspiration or even being lodged in the undercuts of the defect.^9,10^ Therefore, it is important to take care of infant positioning, tray used and the impression material in order to maintain airway patency during impression making.

Filling the defect with a piece of Vaseline gauze helped reduce to minimum any possibilities of impression material lodgement within the defect. The impression compound was softened and placed on an alginate spatula in order to accommodate the small-sized oral cavity. Prone position was essential in keeping the tongue at forward position, avoiding posterior regurgitation of the impression material. Infant crying was satisfactory for ensuring airway patency and elimination of any possibilities of impression material aspiration. The primary impression material was poured with rubber base due to its rapid setting and since the primary cast was only used for construction of the special tray, there was no need for hard dental stone cast.

The special tray was made of self-curing acrylic resin to obtain sufficient rigidity to carry the secondary impression material. In order to obtain proper surface details, the secondary impression was taken with heavy putty type in the current case because of its high viscosity which reduces the aspiration risk. In addition, it reproduces all the areas of interest, while low-viscosity light body was used for improving details of the areas away from the defect without tearing and/or being lodged in the defect.

The final feeding plate was fabricated using fast-setting self-curing acrylic resin in an attempt to construct a single-day feeding appliance to avoid multiple visits. The plate was tied with silk to facilitate easy insertion and removal of the prosthesis and to act as a safety measure to prevent swallowing of the appliance.

The feeding plate was delivered on the same day considering high anxiety of the mother regarding the diminished weight of her infant relative to his age. The normal weight gain of the infant indicated the proper function of the feeding plate.
